# ﻿A new genus *Anamalysia* van Achterberg (Hymenoptera, Braconidae, Alysiinae), six new species, and two new combinations from India, Indonesia, Malaysia, Singapore, Thailand, and Vietnam

**DOI:** 10.3897/zookeys.1126.90916

**Published:** 2022-11-01

**Authors:** Junli Yao, Cornelis van Achterberg, Salmah Yaakop, Khuat Dang Long, Michael J. Sharkey, Eric G. Chapman

**Affiliations:** 1 Biological Control Research Institute, Fujian Agriculture & Forestry University, Fuzhou, Fujian 350002, China Fujian Agricultural & Forestry University Fuzhou China; 2 Naturalis Biodiversity Centre, Postbus 9517, 2300 RA Leiden, Netherlands Naturalis Biodiversity Centre Leiden Netherlands; 3 Department of Biological Sciences and Biotechnology, Faculty of Science and Technology, 43600 National University of Malaysia, Bangi, Selangor, Malaysia National University of Malaysia Selangor Malaysia; 4 Institute of Ecology and Biological Resources, Vietnam Academy of Science and Technology, Cau Giay, Hanoi, Vietnam Institute of Ecology and Biological Resources, Vietnam Academy of Science and Technology Hanoi Vietnam; 5 Department of Entomology, University of Kentucky, Lexington KY 40546-0091, USA University of Kentucky Lexington United States of America

**Keywords:** Alysiini, identification, key, new combination, Oriental, South Asia, taxonomy

## Abstract

A new genus of the tribe Alysiini (Hymenoptera, Braconidae, Alysiinae) is described with specimens from India, Indonesia, Malaysia, Singapore, Thailand, and Vietnam, and six new species are described: *Anamalysiaidiastimorpha***sp. nov.** (type species), *A.knekosoma***sp. nov.**, *A.mellipes***sp. nov.**, *A.transversator***sp. nov.**, *A.vandervechti***sp. nov.**, and *A.vanhengstumi***sp. nov.**. We transfer one species from *Coelalysia* to *Anamalysia*: *A.urbana* (Papp, 1967) **comb. nov.** from Singapore and one species from *Alysiasta* to *Anamalysia*: *A.triangulum* (Fischer, 2006) **comb. nov.** from Malaysia, Laos, Indonesia and Vietnam. A key to the genus of *Anamalysia* is included.

## ﻿Introduction

Alysiini (Hymenoptera, Braconidae, Alysiinae) is a large tribe with 76 genera and over 1565 valid species (Yu et al. 2016). The Alysiini include mostly koinobiont endoparasitoids of cyclorrhaphous dipteran larvae, which use their mandibles (usually with 3 or 4 teeth or lobes) to break open the puparium of the host (Wharton 1977). In this paper, one new genus *Anamalysia* gen. nov., including six new species (*Anamalysiaidiastimorpha* sp. nov. (type species), *A.knekosoma* sp. nov., *A.mellipes* sp. nov., *A.transversator* sp. nov., *A.vandervechti* sp. nov., and *A.vanhengstumi* sp. nov. are described, and two new combinations are reported.

## ﻿Methods

Specimens from Thailand were collected using a Malaise trap in Nakhon Si Thammarat (Namtok Yong National Park) and Doi Chiangdao (the third highest peak in Thailand). Specimens were preserved in 95% ethyl alcohol and then dehydrated using hexamethyldisilazane (**HMDS**) as described in Heraty and Hawks (1998) and subsequently card point mounted. The specimens from India were hand-net collected and kept dry before pinned. Specimens from Malaysia, Indonesia, and Vietnam were collected in 70% alcohol with Malaise traps unless otherwise indicated. The specimens were subsequently prepared according to the AXA method (van Achterberg 2009; van Achterberg et al. 2010) and glued on card points.

For the identification of the subfamily Braconidae, see van Achterberg (1990, 1993), for the terminology and measurements used in this paper, see van Achterberg (1988, 1993), and for additional references, see Yu et al. (2016).

Photographs for species plates were produced using a JVC digital camera mounted on a Leica MZ16 microscope and Auto-Montage stacking software. Photos were slightly processed (cropped and background modified) in Photoshop.

COI sequences of *A.knekosoma* sp. nov. and *A.transversator* sp. nov. are deposited in GenBank. For protocols of DNA extraction, PCR, and sequencing, see Yao et al. (2020). Specimens are deposited in the Oberösterreichisches Landesmuseum, Biologiezentrum, Linz (**BZL**); Institute of Ecology and Biological Resources, Vietnam Academy of Science and Technology, Hanoi, Vietnam (**IEBR**); Naturalis Biodiversity Centre, Leiden, the Netherlands (**RMNH**); Texas A&M University, College Station, Texas, USA (**TAMU**); Universiti Kebangsaan Malaysia, Bangi, Selangor, Malaysia (**UKM**); Queen Sirikit Botanic Gardens Entomology Collection, (**QSBG**) Chiang Mai, Thailand.

## ﻿Results and discussion

### ﻿Taxonomy

#### ﻿ Anamalysia

Taxon classificationAnimaliaHymenopteraBraconidae

van Achterberg gen. nov.

8766F616-87D2-5322-9CEC-251DF9BC2FC9

https://zoobank.org/0A2F1359-EDA4-4A1A-8BC3-31BB43F12139

Figs 1 , 2 , 3 , 4 , 5 , 6 , 7 , 8

##### Type species.

*Anamalysiaidiastimorpha* van Achterberg, sp. nov.

##### Etymology.

From “Anamala (or Anaimala) Hills” (the type locality) and the generic name *Alysia* Latreille, 1804. Anamala or Anaimalai Mountains, also known as the Elephant Mountains, are a range of mountains in the southern Western Ghats of central Kerala (India). Gender: feminine.

##### Diagnosis.

**Figure 1.**
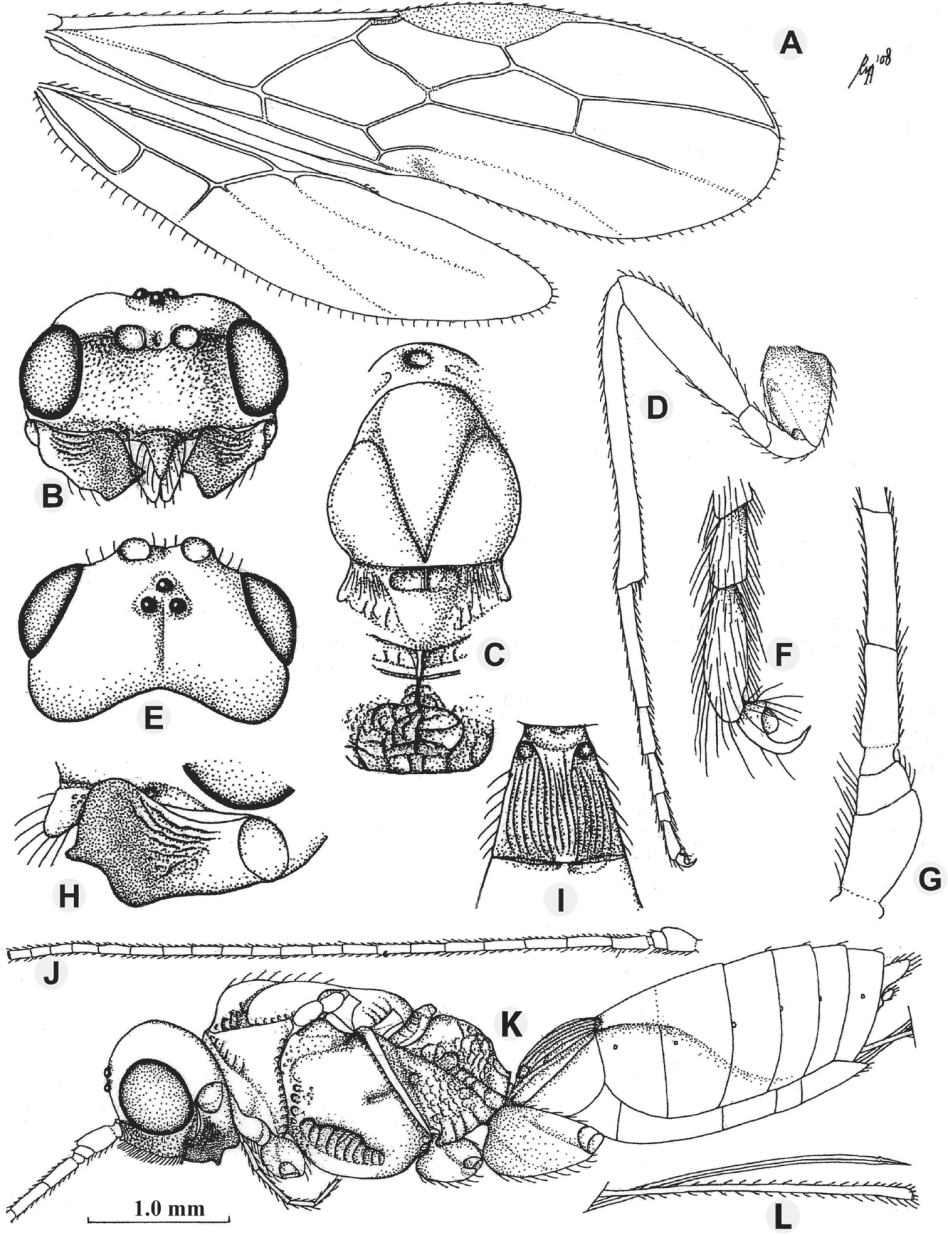
*Anamalysiaidiastimorpha* sp. nov., ♀, holotype **A** wings **B** head, anterior aspect **C** mesosoma, dorsal aspect **D** hind leg **E** head dorsal aspect **F** outer hind claw **G** basal antennal segments **H** mandible full sight on second tooth **I** first metasomal tergite dorsal aspect **J** antenna **K** habitus lateral aspect **L** ovipositor and its sheath. Scale bars: 1.0 mm (**A, D, J–L**); 1.4 mm (**B, C, E, I**); 3.0 mm (**F, G**); 4.0 mm (**H**).

**Figure 2.**
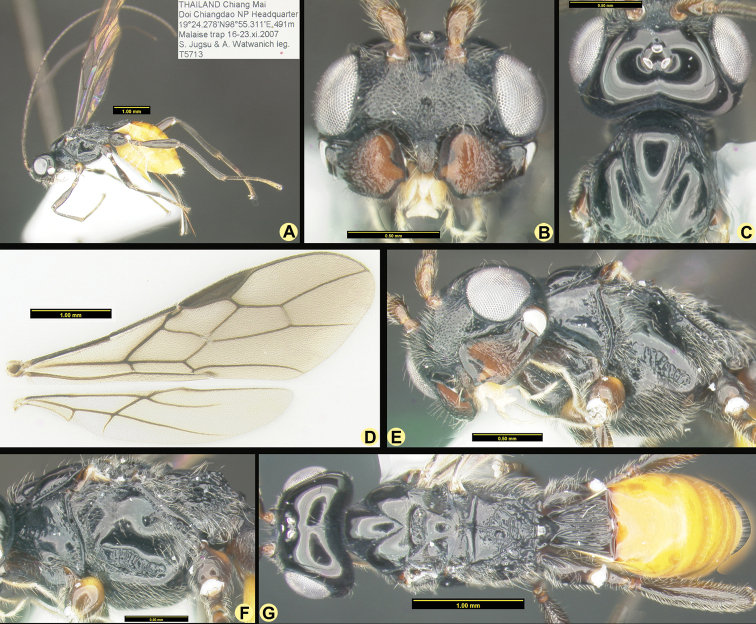
*Anamalysiaknekosoma* sp. nov., ♀, holotype **A** habitus lateral aspect **B** head anterior aspect **C** head and mesoscutum dorsal aspect **D** wings **E** mandible and ventrolateral aspect of head and mesosoma **F** mesosoma lateral aspect **G** habitus, dorsal aspect.

**Figure 3.**
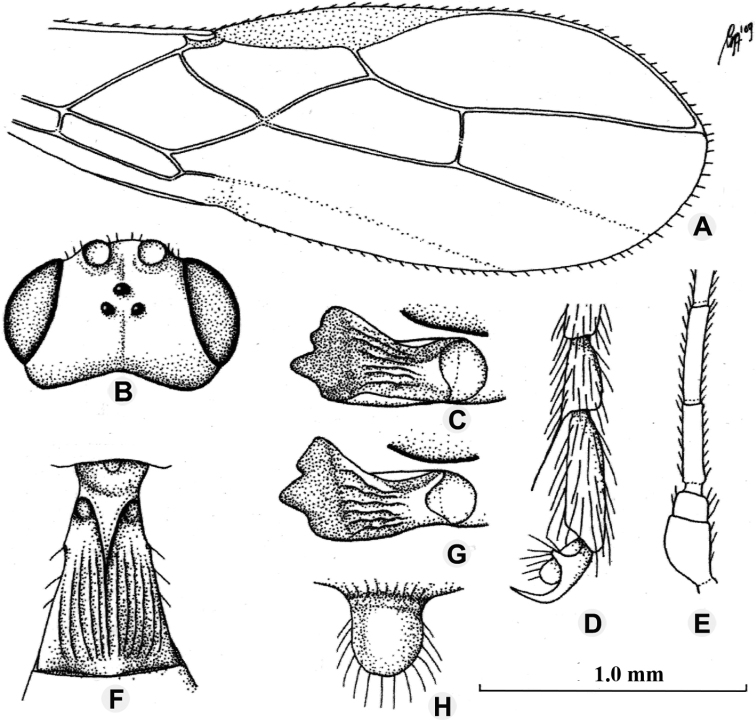
*Anamalysiamellipes* sp. nov., ♀, holotype **A** fore wing **B** head, dorsal aspect **C** mandible, full sight on first tooth **D** outer hind claw **E** basal antennal segments **F** first metasomal tergite, dorsal aspect **G** mandible, full sight on third tooth **H** clypeus. Scale bars: 1.0 mm (**A, B**); 1.5 mm (**C, E–G**); 2.5 mm (**D, H**).

**Figure 4.**
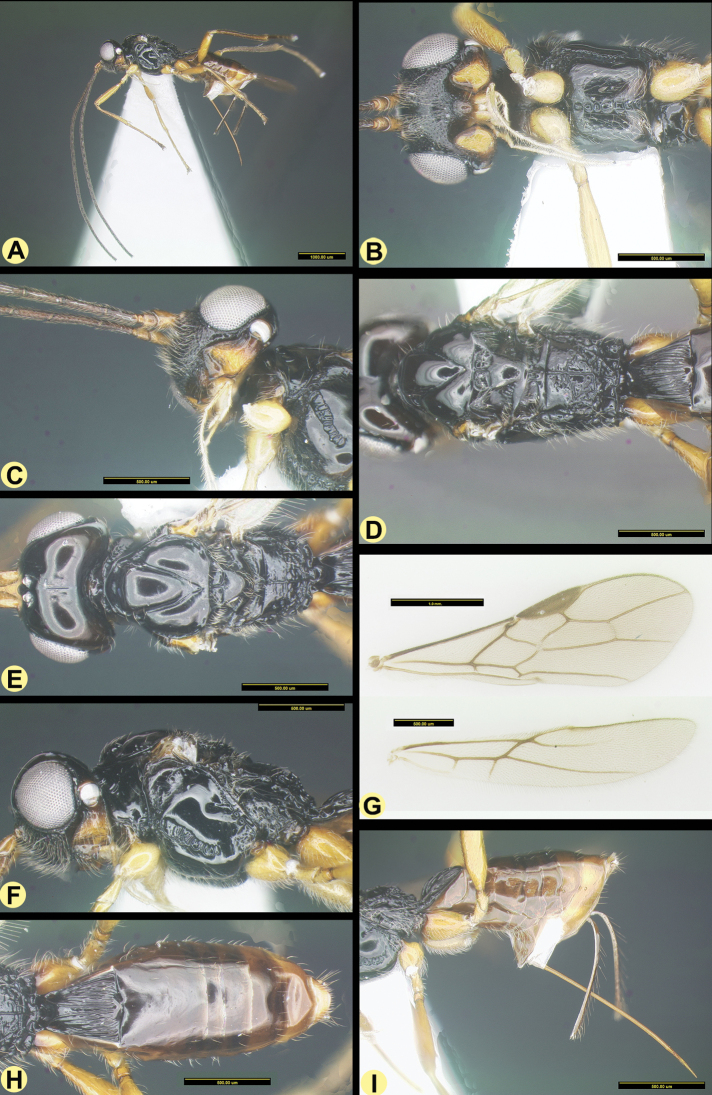
*Anamalysiatransversator* sp. nov., ♀, holotype **A** habitus, lateral aspect **B** head anterior aspect and mesosoma ventrally **C** mandible full sight on first tooth **D** mesosoma full sight on propodeum and first tergite dorsally **E** head and mesosoma dorsal aspect **F** mesosoma lateral aspect **G** wings **H** metasoma dorsal aspect **I** propodeum and metasoma lateral aspect.

**Figure 5.**
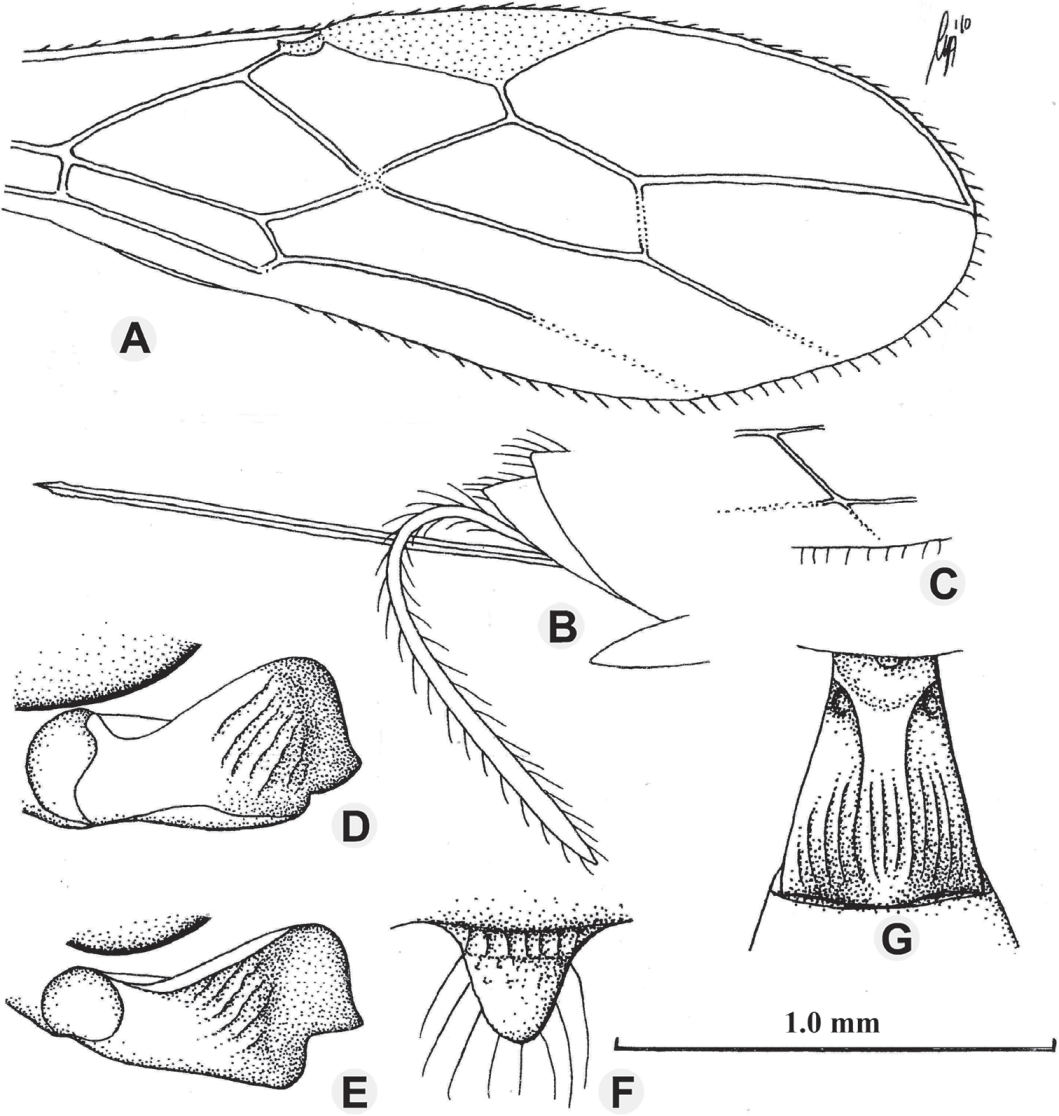
*Anamalysiatriangulum* (Fischer), ♀, holotype **A** fore wing **B** ovipositor and its sheath **C** detail of vein m-cu of hind wing **D** mandible full sight on first tooth **E** mandible full sight on third tooth **F** clypeus **G** first metasomal tergite dorsal aspect. Scale bars: 1.0 mm (**A–C**); 1.5 mm (**D, E**); 1.8 mm (**F**); 1.2 mm (**G**).

**Figure 6.**
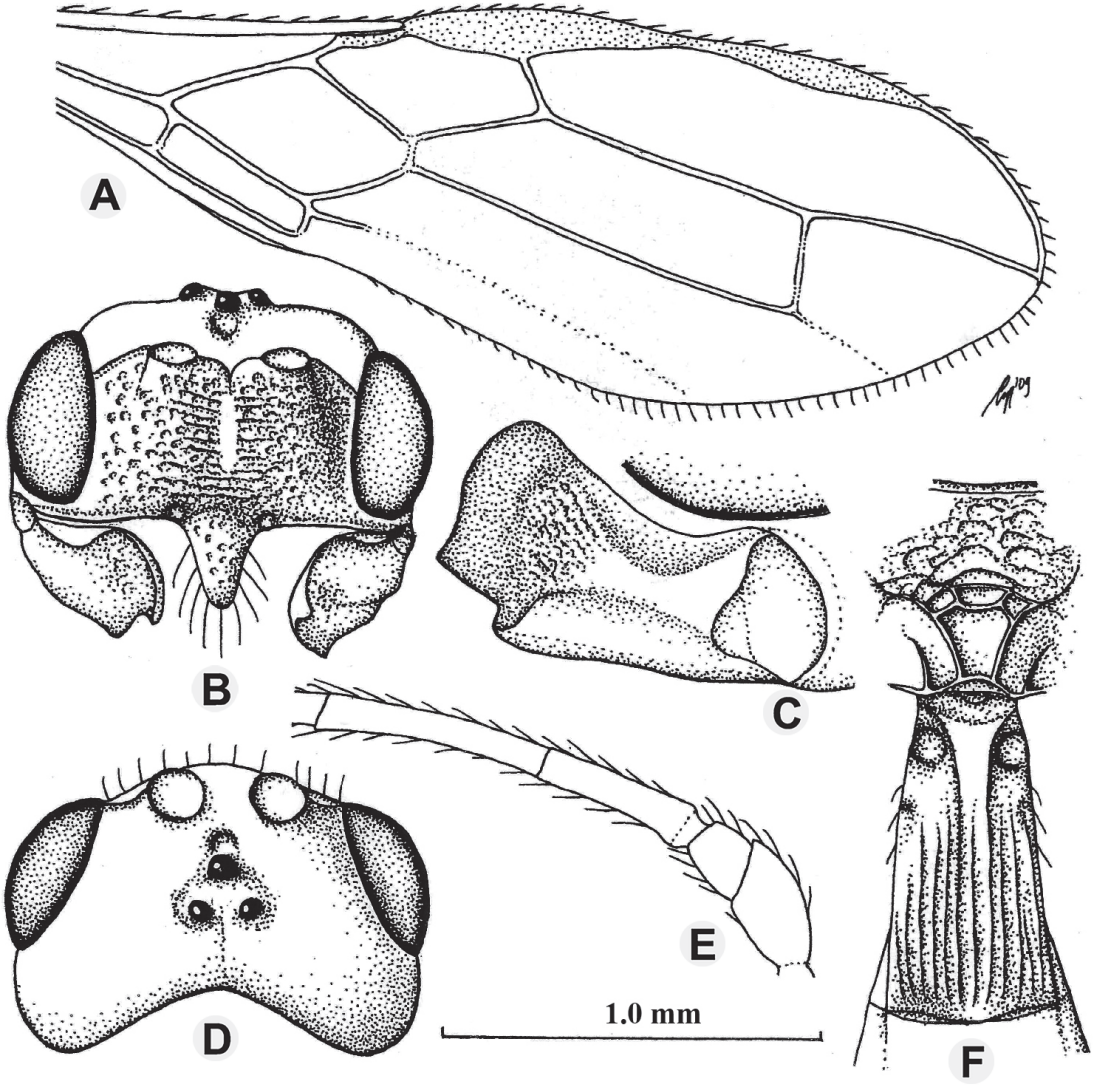
*Anamalysiavandervechti* sp. nov., ♂, holotype **A** fore wing **B** head, anterior aspect **C** mandible, full sight on first tooth **D** head, dorsal aspect **E** basal antennal segment **F** propodeum and first metasomal tergite, dorsal aspect. Scale bars: 1.0 mm (**A**); 1.2 mm (**B, D, F**); 1.8 mm (**C, E**).

Fourth antennal segment 1.1–1.4 times longer than third segment; clypeus rectangularly narrowed ventrally, triangular in dorsal view and with acute ventral apex (Figs 1B, 6B) to round ventral apex (Figs 2B, 3H, 4B, 5F, 8B); area between antennal socket and eye with a narrow groove (Figs 1B, 6B, D, 8B, E); face distinctly sculptured, distinctly transverse and without medio-ventral grooves (Figs 1B, 2B, 4B, 6B, 8B); mandible strongly widened apically and partly sculptured, lateral teeth wide lobe-shaped and second tooth short (Figs 1H, 2E, 3C, G, 4C, 5D, E, 6C, 8G, H); anterior tentorial pits small, far removed from eye; pronope deep and medium-sized to large (Figs 1C, 8D); notauli complete; precoxal sulcus widely crenulate medially and posteriorly narrow or absent; postpectal carina absent; metanotum often distinctly protruding dorsally; vein 2-SR of fore wing 0.9–1.2 times vein 3-SR (for *A.vandervechti*, 2-SR of fore wing 0.5 times vein 3-SR); vein SR1 of fore wing 2.0–2.7 times as long as vein 3-SR (for *A.vandervechti*, vein SR1 of fore wing 0.8 times as long as vein 3-SR); vein r of fore wing much longer than wide; vein m-cu of interstitial or postfurcal; first subdiscal cell of fore wing narrow (Figs 1A, 2D, 3A, 4G, 5A, 8A); vein 3-CU1 of fore wing distinctly longer than vein CU1b (Figs 1A, 2D, 3A, 4G, 5A, 6A, 8A), but slightly shorter in *A.vandervechti* (Fig. 6A); marginal cell of hind wing medium-sized, subparallel-sided; vein 1r-m of hind wing 0.6–0.8 times as long as vein 1- M; vein M+CU of hind wing distinctly longer than vein 1-M; vein m-cu of hind wing distinct, in type species largely sclerotised; tarsal claws rather robust (Figs 1F, 3D); length of first metasomal tergite 1.0–1.4 times its apical width; dorsope present; ovipositor sheath with long erect setae and apically rounded, no apical spine (Figs 1L, 5B), setose part of sheath about 0.4–0.7 times as long as fore wing.

##### Synonymy.

*Alysiasta* Wharton, 1980 sensu Fischer (2006) (partly, not type species); *Coelalysia* Cameron, 1911 sensu Fischer (1988) (partly, not type species).

##### Biology.

Unknown.

##### Distribution.

Oriental.

##### Notes.

The shape of the clypeus is similar to that of the Afrotropical genus *Coelalysia* Cameron, 1911, but *Coelalysia* lacks a complete groove between the antennal socket and the eye, has the dorsope absent or small, the face is largely smooth and strongly transverse, vein M+CU of the hind wing is distinctly shorter than vein 1-M and vein CU1b of fore wing is about as long as vein 3-CU1 or longer, scutellar sulcus about half as long as scutellum, middle tooth of mandible long and mesosternal sulcus narrowly crenulate posteriorly. *Coelalysiaurbana* (Papp, 1967) is excluded from the genus *Coelalysia* and fits well in *Anamalysia*, together with *Alysiastatriangulum* Fischer, 2006 (comb. nov.) and might be the senior synonym of the latter.

### ﻿Key to species of the genus *Anamalysia* gen. nov.

**Table:** 

1	Vein SR1 of fore wing about 0.8 times as long as vein 3-SR (Fig. 6A); vein 3-SR of fore wing about twice as long as vein 2-SR; vein 1-R1 of fore wing of ♂ widened medially (♀ unknown); basal fifth of hind tibia whitish; precoxal sulcus present posteriorly; notauli distinctly narrowly crenulate; length of first tergite about 2.4 times its apical width (Fig. 6F); Indonesia (Sumatra)	***A.vandervechti* van Achterberg, sp. nov.**
–	Vein SR1 of fore wing 2.0–2.8 times as long as vein 3-SR (Figs 1A, 2D, 3A, 4G, 5A, 8A); vein 3-SR of fore wing 0.8–1.2 times as long as vein 2-SR; vein 1-R1 of fore wing of ♀ narrow medially; basal fifth of hind tibia dark brown or yellowish; precoxal sulcus absent posteriorly (Figs 2F, 4F, 8C); notauli smooth; length of first tergite 1.0–1.3 times its apical width (Figs 1I, 2G, 3F, 4H, 5G, 6F, 8F)	**2**
2	Eye in dorsal view 4.2–4.4 times as long as temple and temple narrowed behind eyes (Fig. 3B); middle tooth of mandible free dorsally (Fig. 3C, G); antenna of ♀ with about seven ivory or whitish segments apically; [first tergite 1.4–1.5 times longer than its apical width; hind tibia yellowish brown; lamella above middle tooth of mandible sinuate]; Malaysia	***A.mellipes* van Achterberg & Yaakop, sp. nov.**
–	Eye in dorsal view 1.1–2.6 times as long as temple and temple parallel-sided or widened behind eyes (Figs 1E, 2C, 4E, 8E); middle tooth of mandible connected to curved apical lamella (Figs 1H, 2E, 4C, 5D, E, 8G, H); apical segments of antenna of ♀ black or dark brown (but unknown of *A.urbana*, *A.idiastimorpha*, *A.vanhengstumi*, and *A.transversator*)	**3**
3	Eye in dorsal view 1.1 times longer than temple (Fig. 8E); first tergite about 1.4 times longer than its apical width; temples strongly widened behind eyes (Fig. 8E); hind tibia brownish yellow; [head largely yellowish brown laterally]; Vietnam	***A.vanhengstumi* van Achterberg & Long, sp. nov.**
–	Eye in dorsal view 1.6–2.6 times as long as temple (Figs 1E, 2C, 4E); first tergite 1.0–1.2 times as long as its apical width (Figs 1I, 2G, 4H, 5G); temples parallel-sided behind eyes (Figs 1E, 2C, 4E); hind tibia brown or dark brown, rarely paler	**4**
4	Vein r-m of fore wing subvertical (Fig. 1A); eye in dorsal view 1.6–1.8 times as long as temple (Fig. 1E); clypeus slightly narrower and apically more acute (Fig. 1B); pterostigma dark brown; [second metasomal tergite blackish dorsally and hardly contrasting with black first tergite; vein 3-SR of fore wing 0.8 times as long as vein 2-SR]; India	***A.idiastimorpha* van Achterberg, sp. nov.**
–	Vein r-m of fore wing distinctly inclivous (Figs 2D, 4G, 5A); eye in dorsal view 1.9–3.0 times as long as temple (Figs 2C, 4E); clypeus slightly wider and apically rounded (Figs 2B, 4B, 5F); pterostigma brown	**5**
5	Width of head 2.1–2.4 times medial length (Figs 2C, 4E); vein m-cu of fore wing subinterstitial (Figs 2D, 4G); vein cu-a of fore wing more postfurcal (Figs 2D, 4G)	**6**
–	Width of head 1.7–1.9 times medial length; vein m-cu of fore wing less postfurcal (Fig. 5A); vein cu-a of fore wing subinterstitial (Fig. 5A)	**7**
6	Eye in dorsal view 3.0 times as long as temple (Fig. 4E); propodeum with a complete longitudinal carina, largely smooth anteriorly, except for a short median carina and rugae near it, medially with circular areolate area and posteriorly reticulate, smooth posterior-laterally (Fig. 4D); vein m-cu of hind wing strongly removed from 2-M (Fig. 4G); notauli complete, deep, narrow, and smooth; midpit small and round, connected to notauli (Fig. 4E); length of setose part of ovipositor sheath 0.7 times fore wing and 0.9 times as long as hind tibia (Fig. 4A); length of body 3.5 mm, length of fore wing 3.6 mm	***A.transversator* van Achterberg & Yao, sp. nov.**
–	Eye in dorsal view 2.0 times as long as temple (Fig. 2C); propodeum largely smooth and with sparse punctures anteriorly, except for a short median carina with rugae near it, medially with crown-shaped areolate area and bottom carina protuberant, medio-posteriorly densely reticulate, latero-posteriorly smooth with a longitudinal carina respectively (Fig. 2G); vein m- cu of hind wing interstitial (Fig. 2D); notauli complete, deep, and narrow, smooth, without midpit, more depressed in the end of notauli (Fig. 2C, G); length of setose part of ovipositor sheath as long as fore wing and 0.4 times as long as hind tibia (Fig. 2A); length of body 5.0 mm, length of fore wing 4.6 mm	***A.knekosoma* Yao & van Achterberg, sp. nov.**
7	Length of setose part of ovipositor sheath about 0.44 times as long as fore wing; hind tibia and tarsus yellowish brown, slightly infuscated; mesosoma brown or reddish brown; vein SR1 of fore wing about 2.2 times as long as vein 3-SR (fig. 25 in Papp 1967); vein m-cu of hind wing subinterstitial; Singapore	***A.urbana* (Papp, 1967) comb. nov.**
–	Length of setose part of ovipositor sheath 0.37–0.38 times as long as fore wing; hind tibia (except ivory base) and base of tarsus dark brown or infuscate; mesosoma black or dark chestnut brown; vein SR1 of fore wing 2.3–2.4 times as long as vein 3-SR (Fig. 5A); vein m- cu of hind wing usually distinctly antefurcal; Indonesia, Malaysia, Laos, Vietnam	***A.triangulum* (Fischer, 2006) comb. nov.**

#### ﻿ Anamalysia idiastimorpha

Taxon classificationAnimaliaHymenopteraBraconidae

van Achterberg sp. nov.

C4A10038-43E4-549A-BAF0-5ED643DB433E

https://zoobank.org/839C94E5-28ED-48C2-B96F-CF92048C4DD8

Fig. 1A–L

##### Type material.

***Holotype***, ♀ (RMNH), South India, Anaimalai Hills, Cinchona [plantation?], 3500 ft, v.1964, P. Susai Nathan.

***Non-type***: 1 ♀ (RMNH), India, Kerala, 9–17 km W. Pormudi, 5.xi.1984, B.180, K. Ghorpade.

##### Description.

***Holotype***, ♀, length of body 5.5 mm, length of fore wing 5.0 mm.

***Head*.** Width of head twice its median length, sparsely setose; antenna incomplete, 22+, segments densely setose, length of third segment 0.9 times as long as fourth segment, length of third and fourth segments 3.0 and 4.6 times their width, respectively (Fig. 1G, J); length of maxillary palp 1.2 times height of head; eye in dorsal view 1.8 times as long as temple; temple in dorsal view subparallel-sided behind eyes (Fig. 1E); OOL: diameter of ocellus: POL = 24:7:6 (Fig. 1E); frons flat medially and convex laterally, smooth and with pit between antennal sockets; antennal sockets distinctly protruding; smooth narrow groove between antennal sockets and eye; minimum width of face 0.55 times maximum width of head, densely punctate submedially, more sparsely on remainder of face, with long setae, without crenulate grooves ventrally (Fig. 1B); clypeus narrow, triangular, with long setae and ventrally acute and its surface with a few punctures, moderately convex dorsally, length of malar space 0.1 times basal width of mandible; mandible coarsely rugose medially, strongly widened apically, its medial length 1.4 times its maximum width, upper tooth large and truncate lobe-shaped, with ventral tooth rounded and lobe-shaped, connected to a ventral carina (Fig. 1H).

***Mesosoma*.** Length of mesosoma 1.6 times its height; pronotum dorsally with large deep and round dorsope; side of pronotum with some coarsely crenulate antero-medially, posteriorly finely crenulate and remainder smooth; epicnemial area dorsally punctulate, medially crenulate and ventrally punctate; precoxal sulcus absent posteriorly, remainder very wide and coarsely crenulate; remainder of mesopleuron smooth; episternal scrobe linear; pleural sulcus finely crenulate, but ventrally more coarsely crenulate (Fig. 1K); mesosternal sulcus coarsely crenulate, rather wide posteriorly; metapleuron largely coarsely rugose-punctate; notauli complete, deep and narrow, mainly smooth (Fig. 1C); mesoscutum strongly shiny and largely glabrous, but with long setae near notauli and lateral carina; mesoscutum without a medio- posterior depression; axilla conspicuously setose and lateral carina lamelliform protuberant; scutellar sulcus deep, with one carina and some punctures, 0.3 times as long as scutellum; scutellum rather convex in lateral view; metanotum distinctly lamelliform protruding dorsally in lateral view; surface of propodeum largely smooth antero-laterally, remainder vermiculate- reticulate, without areola and with long irregular median carina; propodeal spiracle round, small and in front of middle of propodeum.

***Wings*.** Pterostigma subelliptical (Fig. 1A), its posterior margin hardly curved; vein r issued distinctly behind middle of pterostigma and distinctly oblique; r:3-SR:SR1 = 5:20:53; 1-SR+M sinuate (left wing) or straight (right wing); SR1 straight; cu-a short and oblique, postfurcal; 1- CU1:2-CU1 = 1:14; 2-SR:3-SR:r-m = 24:20:10; m-cu distinctly postfurcal, slightly converging to 1-M posteriorly; first subdiscal cell 4.7 times as long as wide; CU1b distinctly shorter than 3-CU1 and 3-CU1 oblique. Hind wing: M+CU:1-M:1r-m = 30:22:18; m-cu distinctly developed and removed from 2-M and largely sclerotised (Fig. 1A).

***Legs*.** Outer side of hind coxa finely punctate and densely setose, dorsally smooth; middle coxa strongly protruding forwards ventrally, less so of hind coxa; tarsal claws moderately robust; length of femur, tibia, and basitarsus of hind leg 3.7, 11.7, and 7.5 times their width, respectively; hind tibia and basitarsus rather short and adpressed bristly setose (Fig. 1D, F).

***Metasoma*.** Length of first tergite equal to its apical width, its surface regularly costate-striate, its dorsal carinae complete (Fig. 1I); laterope absent; dorsope large and deep; remainder of metasoma smooth and rather depressed; ovipositor with minute dorsal notch and some ventral teeth; length of setose part of ovipositor sheath 0.39 times fore wing and nearly as long as hind tibia; apex of ovipositor sheath subtruncate and no apical spine (Fig. 1L); hypopygium medium- sized and apically acute in lateral view (Fig. 1K).

***Colour*.** Black; scapus, pedicellus, and tegulae more or less chestnut brown; palpi pale yellowish; metasoma (except black first tergite and dark parts of second and third tergites) and apically femur and tibia of fore leg brownish yellow; remainder of antenna (as far as present) and of legs, mandible, second tergite dorsally and middle of third tergite, ovipositor sheath, pterostigma (but apex paler brown), and most veins dark brown; wing membrane slightly infuscate.

***Variation*.** The specimen from Kerala is very similar but has the pterostigma narrower, third antennal segment comparatively slender (3.5 times longer than wide and about as wide as fourth segment) and vein m-cu of hind wing unsclerotised. It has 46 antennal segments, 1.8 times as long as fore wing and its apical half completely black; length of fore wing 4.2 mm and of body 4.6 mm; eye in dorsal view 1.7 times as long as temple; length of setose part of ovipositor sheath 0.37 times fore wing.

##### Distribution.

South India (Anamalai Hills).

##### Etymology.

Named after the generic name *Idiasta* Foerster,1863 because of its morphological similarity.

#### ﻿ Anamalysia knekosoma

Taxon classificationAnimaliaHymenopteraBraconidae

van Achterberg & Yao sp. nov.

A7A0FDD9-AAF2-5F92-A82A-DDB58EA5D0C9

https://zoobank.org/9E4DD0CE-ED51-42B1-9A15-5BC0FF97B55E

Fig. 2A–G

##### Type material.

***Holotype***, ♀ (QSBG), Thailand, Chiang Mai, Doi Chiangdao NP Headquarters 19°24.3'N, 98°55.3'E, 491 m, Malaise trap 16–23.xi.2007, S. Jugsu & A. Watwanich leg. T5713, GenBank accession number MG912777 (COI).

##### Description.

***Holotype***, ♀, length of body 5.0 mm, length of fore wing 4.6 mm.

***Head*.** Width of head 2.4 times its median length, sparsely setose and strongly shiny; antenna complete (Fig. 2A), 47-segmented, segments densely setose, length of third segment 0.7 times as long as fourth segment, length of third and fourth segments 3.8 and 6.5 times their width, respectively; length of maxillary palp 1.5 times height of head; eye in dorsal view 2.0 times as long as temple; temple in dorsal view subparallel-sided behind eyes (Fig. 2C); OOL: diameter of ocellus: POL = 14:6:11; frons flat medially (except an incomplete median groove, anteriorly half deep groove and posteriorly half with groove trace) and convex laterally, smooth; antennal sockets distinctly protruding; with a smooth, narrow and superficial groove between antennal sockets and eye; minimum width of face 0.35 times maximum width of head, densely rugulose- punctate, with a Y-shaped carina medially (from antenna sockets to clypeus), medio-posteriorly with fine reticulate, with rather dense and long setae (Fig. 2B); clypeus wide, triangle, width 1.1 times its length, with long setae and ventrally rounded and its surface largely smooth except a few punctures (Fig. 2B); length of malar space 0.1 times basal width of mandible; mandible sparsely rugose medially except teeth part smooth, strongly widened dorsally, its medial length 1.8 times its maximum width, upper tooth large and truncate lobe-shaped, with ventral tooth rather small, rounded and lobe-shaped, connected to a carina (Fig. 2E).

***Mesosoma*.** Length of mesosoma 1.6 times its height; pronotum dorsally with large deep and round dorsope; side of pronotum with some coarse crenulate anteriorly, posteriorly finely crenulate and remainder smooth (Fig. 2F); epicnemial area dorsally smooth, medially crenulate and ventrally punctate-rugose; precoxal sulcus anterior 1/5 smooth and remainder crenulate, widely crenulate anteriorly, narrowed after its middle and absent posteriorly (Fig. 2F); remainder of mesopleuron smooth; episternal scrobe round, deep; pleural sulcus anteriorly smooth and punctulate, with dense setae, posteriorly coarsely crenulate; mesosternal sulcus coarsely crenulate, rather wide posteriorly; metapleuron largely smooth and punctulate, with fine reticulate ventrally; notauli complete, deep and narrow, smooth, without midpit, more depressed in the end of notauli (Fig. 2G); mesoscutum strongly shiny and largely glabrous, but with some long setae near notauli and lateral carina and medial lobe protuberant; mesoscutum without a separate medio-posterior depression (Fig. 2G); axilla rather setose and lateral carina moderately protuberant; scutellar sulcus deep, with one carina and coarsely rugae, without punctures, 0.2 times as long as scutellum; scutellum rather convex in lateral view; metanotum distinctly lamelliform protruding posteriorly in lateral view; propodeum largely smooth and with sparse punctures anteriorly, except for a short median carina with rugae near it, medially with crown-shaped areolate area and bottom carina protuberant, medio-posteriorly densely reticulate, latero-posteriorly smooth with a longitudinal carina respectively (Fig. 2G); propodeal spiracle round, small and medially at propodeum.

***Wings*.** Pterostigma subelliptical, its posterior margin hardly curved; vein r issued distinctly behind middle of pterostigma and distinctly oblique; r:3-SR:SR1 = 7:22:50; 1-SR+M rather sinuate; SR1 straight, slightly curved posteriorly; cu-a short and oblique, strongly postfurcal; 2-SR:3-SR:r-m = 38:36:19, vein r-m of fore wing distinctly inclivous; m-cu slightly postfurcal, slightly converging to 1-M posteriorly; first subdiscal cell 5.0 times as long as wide; 3- CU1:CU1b = 3.2 and 3-CU1 oblique. Hind wing: M+CU:1-M:1r-m = 83:53:45; m-cu distinctly developed and interstitial (Fig. 2D).

***Legs*.** Outer side of hind coxa largely smooth, punctulate and moderately setose, dorsally shiny and smooth; middle coxa strongly protruding forwards ventrally, hind coxa gradually narrowed; tarsal claws moderately robust; length of femur, tibia and basitarsus of hind leg 3.9, 10.0, and 6.9 times their width, respectively; middle tibia and basitarsus rather short and adpressed setose (Fig. 2A).

***Metasoma*.** Length of first tergite 1.1 times its apical width, its surface regularly costate-striate, its dorsal carinae complete and united submedially (Fig. 2G); laterope absent; dorsope large and deep; remainder of metasoma smooth and rather depressed; ovipositor with minute dorsal notch and some ventral teeth; setose part of ovipositor sheath as long as fore wing and 0.4 times as long as hind tibia (Fig. 2A); apex of ovipositor sheath subtruncate and no apical spine; hypopygium medium-sized and apically acute in lateral view (Fig. 2A).

***Colour*.** Black; head and first tergite apically chestnut brown; scapus, pedicellus, and mandible apically brownish yellow; tegulae brown, but dorsal half brownish yellow; fore leg light brown (except coxa brownish yellow with yellow spot basally, trochanter, trochantellus with dark yellow spot apically, tibia basally and tarsus apically darkened yellow); middle leg light chestnut brown (but coxa brown with dark yellow spot basally, trochanter, trochantellus with dark yellow spot apically, tarsus apically light brown); hind leg chestnut brown as head (but coxa with brown spot basally, trochanter, trochantellus similar as middle leg, tarsus apically light brown); palpi pale yellowish; remainder of antenna, mandible basally, ovipositor sheath dark brown, metasoma (except blackish first tergite) yellowish brown dorsally, apical segment and metasoma ventrally and laterally yellow (Fig. 2I); pterostigma and most veins brown; wing membrane subhyaline.

##### Distribution.

Thailand.

##### Etymology.

Named after the mainly conspicuously yellow metasoma of the holotype; “knekos” is Greek for yellow and “soma” is Greek for body.

#### ﻿ Anamalysia mellipes

Taxon classificationAnimaliaHymenopteraBraconidae

van Achterberg & Yaakop sp. nov.

55C17407-DBE7-51D7-9C78-CCBB01DE4221

https://zoobank.org/50584F60-BECC-4E9D-A6CF-2ECB4A7F2B6F

Fig. 3A–H

##### Type material.

***Holotype***, ♀ (RMNH), Malaysia, SW Sabah, near Long Pa Sia (West), c. 1050 m, 25.xi–8.xii.1987, Mal. trap 3, C. v. Achterberg, RMNH’87, DNA voucher number “94”. ***Paratypes*** (5 ♀): 1 ♀ (TAMU), Indonesia, West Kalimantan, Gunung Palung Nat. Park, 15.vi–15.viii.1991, Darling, Sutrisno & Rosichon, IIS 910122; Cabang Panti Res. Station, 1° [= primary] rainforest, 100–400 m, alluvial-light gap, 1°15'S, 110°5'E, Malaise trap, head; 1 ♀ (UKM), Malaysia, N. Sembilan, Pasoh Forest Reserve, 24.x.2002, (50 ha plot), Ng, Y.F. & Ruslan, DNA voucher number “84”; 1 ♀ (UKM), [West Malaysia], Johor: Endau-Rompin Selai, 25.ix–1.x.2004, Shah, Roslan, Fauzi, DNA voucher number “59”; 1 ♀ (RMNH), W. Malaysia, Johor, Endau Rompin, Kampung Peta, ix.2007, Mal. trap, Ruslan, Fauzi & Norlie; 1 ♀ (RMNH), W. Malaysia, Pahang, Hutan Kuala Lompat, 29.xii.2006–13.i.2007, Mal. Tr., Ruslan, Fauzi & Norlie, DNA voucher number “73”.

##### Description.

***Holotype***, ♀, length of body 3.6 mm, length of fore wing 3.1 mm.

***Head*.** Width of head 1.7 times its median length, sparsely setose and strongly shiny; antenna incomplete, 24+, segments densely setose, length of third segment 0.9 times as long as fourth segment, length of third and fourth segments 4.0 and 4.3 times their width, respectively (Fig. 3E); length of maxillary palp of paratypes 1.4 times height of head (missing in holotype); eye in dorsal view 4.4 times as long as temple; temple in dorsal view subparallel-sided behind eyes (Fig. 3B); OOL: diameter of ocellus: POL = 11:4:3; frons flat medially (except a complete median groove) and convex laterally, smooth and no pit between antennal sockets; antennal sockets distinctly protruding; with a smooth, narrow and superficial groove between antennal sockets and eye; minimum width of face 0.5 times maximum width of head, densely rugulose-punctate submedially, more sparsely on remainder of face and transversely rugose ventrally and smooth medially, with rather long setae, without crenulate grooves ventrally; clypeus narrow, nearly parallel-sided, with long setae and ventrally rounded and its surface largely smooth except a few punctures (Fig. 3H); length of malar space 0.1 times basal width of mandible; mandible rugose medially, strongly widened dorsally, its medial length 1.5 times its maximum width, upper tooth large and truncate lobe-shaped, with ventral tooth rather small, rounded and lobe-shaped, connected to a carina (Fig. 3C, G).

***Mesosoma*.** Length of mesosoma 1.5 times its height; pronotum dorsally with large deep and round dorsope; side of pronotum with some coarse crenulae anteriorly and medially, posteriorly finely crenulate and remainder smooth; epicnemial area dorsally smooth, medially crenulate and ventrally punctate-rugose; precoxal sulcus widely crenulate anteriorly, narrowed after its middle and absent posteriorly; remainder of mesopleuron smooth; episternal scrobe round, deep; pleural sulcus coarsely crenulate; mesosternal sulcus coarsely crenulate, rather wide posteriorly; metapleuron largely smooth, with some rugae ventrally; notauli complete, deep, narrow, and smooth; mesoscutum strongly shiny and largely glabrous, but with some long setae near notauli and lateral carina and medial lobe protuberant; mesoscutum without a separate medio-posterior depression; axilla rather setose and lateral carina moderately protuberant; scutellar sulcus deep, with one carina and no punctures, 0.4 times as long as scutellum; scutellum rather convex in lateral view; metanotum distinctly lamelliform protruding posteriorly in lateral view; surface of propodeum largely smooth anteriorly, except for a short median carina, medially with wide triangular areolate area and posteriorly reticulate; propodeal spiracle round, small and submedially at propodeum.

***Wings*.** Pterostigma subelliptical (Fig. 3A), its posterior margin hardly curved; vein r issued distinctly behind middle of pterostigma and distinctly oblique; r:3-SR:SR1 = 5:21:53; 1-SR+M rather sinuate; SR1 straight; cu-a short and oblique, interstitial; 2-SR:3-SR:r-m = 25:21:13; m- cu slightly postfurcal, slightly converging to 1-M posteriorly; first subdiscal cell 6.5 times as long as wide; CU1b distinctly shorter than 3-CU1 and 3-CU1 oblique. Hind wing: M+CU:1- M:1r-m = 30:35:13; m-cu distinctly developed and removed from 2-M.

***Legs*.** Outer side of hind coxa largely smooth, punctulate and moderately setose, dorsally shiny and smooth; middle coxa strongly protruding forwards ventrally, hind coxa gradually narrowed; tarsal claws moderately robust (Fig. 3D); length of femur, tibia, and basitarsus of hind leg 4.2, 14.0, and 10.3 times their width, respectively; hind tibia and basitarsus rather short and adpressed setose.

***Metasoma*.** Length of first tergite 1.4 times its apical width, its surface regularly costate-striate, its dorsal carinae nearly complete and united submedially (Fig. 3F); laterope absent; dorsope large and deep (Fig. 3F); remainder of metasoma smooth and rather depressed; ovipositor with minute dorsal notch and some ventral teeth; length of setose part of ovipositor sheath 0.34 times fore wing and 0.8 times as long as hind tibia; apex of ovipositor sheath subtruncate and no apical spine; hypopygium medium-sized and apically acute in lateral view.

***Colour*.** Dark chestnut brown; scapus, pedicellus, and tegulae more or less brown; palpi pale yellowish; metasoma (except first tergite and base of second tergite), remainder of antenna (as far as present), mandible, coxae (but paler apically) and ovipositor sheath brown; two basal segments of hind tarsus darkened; remainder of legs brownish yellow; pterostigma and most veins pale brown; wing membrane subhyaline.

***Variation*.** Length of fore wing 2.8–3.1 mm and of body 3.3–3.6 mm; antenna of ♀ with 36 (1) segments, 1.9 times as long as fore wing and seven or eight apical segments white or ivory; vein SR1 of fore wing 2.5–3.1 times vein 3-SR; length of first tergite 1.4–1.5 times its apical width; eye in dorsal view 4.2–4.4 times as long as temple; length of setose part of ovipositor sheath 0.32–0.35 times fore wing.

##### Distribution.

Malaysia (East Malaysia: Sabah; West Malaysia: Johor, Pahang, Sembilan).

##### Etymology.

Named after its largely brownish yellow legs; “mel, mellis” is Latin for honey, and “pes, pedus” is Latin for leg.

#### ﻿ Anamalysia transversator

Taxon classificationAnimaliaHymenopteraBraconidae

Yao & van Achterberg sp. nov.

29A6F56B-6A26-568C-8455-07EFF5FB1D32

https://zoobank.org/F99CC0C6-2493-4D43-8EC8-E211FF121F00

Fig. 4A–I

##### Type material.

***Holotype***, ♀ (QSBG), Thailand, Nakhon Si Thammarat, Namtok Yong NP TV aerial, 8°14.3'N, 99°48.3'E, 952 m, Malaise trap, 26.i–2.ii.2009, Paiboon leg. T4307, Genbank accession number MG912720 (COI).

##### Description.

***Holotype***, ♀, length of body 3.5 mm, length of fore wing 3.6 mm.

***Head*.** Width of head 2.1 times its median length, sparsely setose and strongly shiny; antenna incomplete, 26+, however, longer than body (Fig. 4A), segments densely setose, length of third segment 0.7 times as long as fourth segment, length of third and fourth segments 4.3 and 8.0 times their width, respectively; length of maxillary palp 1.5 times height of head; eye in dorsal view 3.0 times as long as temple; temple in dorsal view subparallel-sided behind eyes (Fig. 4E); OOL: diameter of ocellus: POL = 27:6:7; frons flat medially (except an incomplete median groove, anterior half with deep groove and posterior half with groove trace) and convex laterally, smooth; antennal sockets distinctly protruding; with a smooth, narrow, and superficial groove between antennal sockets and eye; minimum width of face 0.6 times maximum width of head, densely rugulose-punctate, with a Y-shaped carina medially (from antenna sockets to clypeus), anterior 2/3 (between and along Y-shaped carina) medially smooth, with rather dense and long setae (Fig. 4B); clypeus wide, triangular, width 2.0 times its length, with long setae and ventrally rounded and its surface largely smooth except a few punctures (Fig. 4B); length of malar space 0.1 times basal width of mandible; mandible sparsely rugose medially except teeth part smooth, strongly widened dorsally, its medial length 2.0 times its maximum width, upper tooth large and truncate lobe-shaped, with ventral tooth rather small, rounded and lobe-shaped, connected to a carina (Fig. 4C).

***Mesosoma*.** Length of mesosoma 1.3 times its height; pronotum dorsally with large deep and round dorsope (Fig. 4E); side of pronotum with some coarse crenulate anteriorly, posteriorly finely crenulate and remainder smooth; epicnemial area dorsally smooth, medially crenulate and ventrally punctate-rugose; precoxal sulcus anterior 1/5 smooth and remainder crenulate, widely crenulate anteriorly, narrowed after its middle and absent posteriorly (Fig. 4F); remainder of mesopleuron smooth; episternal scrobe round, deep; pleural sulcus anteriorly smooth and punctulate, with dense setae, posteriorly coarsely crenulate; mesosternal sulcus coarsely crenulate, rather wide posteriorly; metapleuron largely smooth, with some rugae dorsally and ventrally; notauli complete, deep, narrow. and smooth; midpit small and round, connected to notauli (Fig. 4D, E); mesoscutum strongly shiny and largely glabrous, but with some long setae near notauli and lateral carina and medial lobe protuberant; mesoscutum without a separate medio-posterior depression; axilla rather setose and lateral carina moderately protuberant; scutellar sulcus deep, with one carina and coarsely rugae, without punctures, 0.4 times as long as scutellum (Fig. 4D, E); scutellum rather convex in lateral view; metanotum distinctly lamelliform protruding posteriorly in lateral view; propodeum with a complete longitudinal carina, largely smooth anteriorly, except for a short median carina and rugae near it, medially with circular areolate area and posteriorly reticulate, smooth latero-posteriorly (Fig. 4D, E); propodeal spiracle round, small and medially at propodeum.

***Wings*.** Pterostigma subelliptical (Fig. 4G), its posterior margin hardly curved; vein r issued distinctly behind middle of pterostigma and distinctly oblique; r:3-SR:SR1 = 14:49:103; 1- SR+M rather sinuate; SR1 straight, slightly curved posteriorly; cu-a short and oblique, strongly postfurcal; 2-SR:3-SR:r-m = 30:29:14,vein r-m of fore wing distinctly inclivous; m-cu slightly postfurcal, slightly converging to 1-M posteriorly; first subdiscal cell 3.8 times as long as wide; 3-CU1:CU1b = 3.2 and 3-CU1 oblique. Hind wing: M+CU:1-M:1r-m = 29:23:10; m-cu distinctly developed and removed from 1r-m.

***Legs*.** Outer side of hind coxa largely smooth, punctulate and moderately setose, dorsally shiny and smooth; middle coxa strongly protruding forwards ventrally, hind coxa gradually narrowed; tarsal claws moderately robust; length of femur, tibia, and basitarsus of hind leg 4.3, 10.0, and 8.7 times their width, respectively; middle tibia and basitarsus rather short and adpressed setose (Fig. 4A).

***Metasoma*.** Length of first tergite 1.0 times its apical width, its surface regularly costate-striate, its dorsal carinae nearly complete and united submedially (Fig. 4H); laterope absent; dorsope large and deep (Fig. 4H); remainder of metasoma smooth and rather depressed; ovipositor with minute dorsal notch and some ventral teeth; length of setose part of ovipositor sheath 0.7 times fore wing and 0.9 times as long as hind tibia; apex of ovipositor sheath subtruncate and no apical spine; hypopygium medium-sized and apically acute in lateral view (Fig. 4I).

***Colour*.** Black; head and first tergite chestnut brown; remainder of metasoma yellow; scapus, pedicellus, mandible apically, tegulae, and middle and hind legs (except tibia and tarsus brown, three apical tarsus lightened) brownish yellow; palpi pale yellowish; fore leg yellow (but apical tarsus more or less brown); remainder of antenna (as far as present), mandible basally and ovipositor sheath dark brown; pterostigma and most veins brown; wing membrane subhyaline.

##### Distribution.

Thailand.

##### Etymology.

Named after the comparatively transverse head in dorsal view (Fig. 4E).

#### ﻿ Anamalysia triangulum

Taxon classificationAnimaliaHymenopteraBraconidae

(Fischer, 2006) comb. nov.

48310EA9-61D9-5517-B438-4E3EDBFEBDD3

Fig. 5A–G

Alysiasta triangulum Fischer, 2006: 612–613.

##### Type material.

***Holotype***, ♀ (BZL), Malaysia, Pahang, 30 km NE Raub, ~ 300 m, Lata Lembik, iv–v.2002, ET [electric grid trap], 3°56'N, 101°38'E, E. Jendek & O. Šauša leg., “Holotype, ♀ *Alysiastatriangulum* sp. n., det. Fischer, 2005”.

##### Additional material.

2 ♀ (TAMU, RMNH), Indonesia, Sumatra, Aceh, Gunung Leuser Nat. Park, Ketambe Res. Station, 1–30.xi.1989, per D.C. Darling, IIS 890010, 1° [= primary] rainforest, young forest, terrace 3, closed canopy, 350 m, 3°41'N, 97°39'E, Malaise trap w/ pans; 1 ♀ (RMNH), Malaysia, SW Sabah, near Long Pa Sia (West), c. 1020 m, 25.xi– 8.xii.1987, Mal. trap 2, C. v. Achterberg, RMNH’87, DNA voucher number “63”; 1 ♂ (RMNH), “13”, Indonesia, Sumatra, Fort de Kock [= Bukittinggi], x.1913, Edw. Jacobson; 1 ♀ (IEBR), “Alysi. 029”, VN [= Vietnam], Ninh Binh, Cuc Phuong N.P., 7–9 v.2002, Kh.D. Long”.

##### Notes.

Length of the hind femur of the holotype is four times its width, not five times as indicated in the original description; length of the first metasomal tergite 1.1 times its apical width (Fig. 5G), not 1.3 times as mentioned in the original description; the eye in dorsal view 1.9 times as long as the temple; the hind tibia (except ivory base) and base of the hind tarsus dark brown; vein SR1 of the fore wing 2.4 times as long as vein 3-SR (Fig. 5A). Colour of head and of mesosoma varies from nearly black to chestnut brown. The male from Sumatra and the female from Sabah have the metasoma dark brown and vein m-cu of hind wing unsclerotised basally (as in holotype).

##### Distribution.

Malaysia (West), Laos, Indonesia, Vietnam. The latter two are new country records for this species.

#### ﻿ Anamalysia urbana

Taxon classificationAnimaliaHymenopteraBraconidae

(Papp, 1967) comb. nov.

622181DA-3E2D-5A0C-BE3E-DE43EE64F7FC

Phaenocarpa urbana Papp, 1967: 152–154.
Coelalysia urbana ; Fischer, 1988: 116–118 (redescription).

##### Distribution.

Singapore.

##### Notes.

The two existing descriptions are rather confusing. In the original description the first tergite is 1.1 times longer than its apical width, but according to the redescription by Fischer (1988), it is 1.2 times (in the text) or 1.3–1.4 times (in his fig. 39). Vein r-m of fore wing is strongly inclivous according to the original description (Papp 1967: fig. 25) and only moderately so in Fischer (1988: fig. 38). If the original description is accepted then *A.urbana* is hardly separable from *A.triangulum* and the latter might be well a junior synonym of *A.urbana* when more specimens become available. The difference in colour may be the result of ageing and exposure to sunlight.

#### ﻿ Anamalysia vandervechti

Taxon classificationAnimaliaHymenopteraBraconidae

van Achterberg & Yaakop sp. nov.

D3A69F99-F5D7-5766-A829-1CFCAEE313C2

https://zoobank.org/6B584A68-F8EB-4810-B91A-2221A3AF557A

Fig. 6 A–F

##### Type material.

***Holotype***, ♂ (RMNH), Museum Leiden, [Indonesia], N.O. Sumatra, Deli, Sibolangit, 4.i.1955, J. v. d. Vecht.

##### Description.

***Holotype***, ♂, length of body 4.1 mm, length of fore wing 3.5 mm.

***Head*.** Width of head 1.9 times its median length, largely glabrous dorsally; antenna incomplete, with short adpressed setae and six basal strongly shiny, length of third segment 0.8 times as long as fourth segment, length of third and fourth segments 4.7 and 6.0 times their width, respectively (Fig. 6E); length of maxillary palp 1.3 times height of head; eye in dorsal view 1.5 times as long as temple; temple in dorsal view subparallel-sided (Fig. 6D); OOL: diameter of ocellus: POL = 14:3:4 (Fig. 6D); minimum width of face 0.6 times maximum width of head and 1.7 times its height, coarsely punctate, weakly convex, with long setae and medio-ventrally densely rugose; with oblique groove from antennal socket to eye (Fig. 6B); clypeus elongate and narrow (Fig. 6B), sparsely punctate; vertex strongly shiny, weakly convex, and depressed near stemmaticum; anterior tentorial pit small, round, and far from eye (Fig. 6B); length of malar space 0.1 times basal width of mandible; mandible strongly widened, 1.4 times as long as wide, subapically partly coarsely punctate-rugose, first tooth broadly lobe-shaped, and continuous with minute tooth and separated from third medium-sized tooth (Fig. 6C).

***Mesosoma*.** Length of mesosoma 1.5 times its height; pronotum with medium-sized pronope; antescutal depression absent; side of pronotum largely crenulate medially; epicnemial area distinctly crenulate; precoxal sulcus complete, wide and coarsely crenulate; remainder of mesopleuron smooth; episternal scrobe large, deep, and round; pleural sulcus largely smooth dorsally and distinctly crenulate ventrally; mesosternal sulcus wide and coarsely crenulate posteriorly and narrowed anteriorly; metapleuron coarsely reticulate; notauli complete, deeply impressed, rather narrow but distinctly crenulate; medio-posterior depression absent; mesoscutum smooth, glabrous, and its lateral carina interrupted in front of tegulae; scutellar sulcus deep, about twice as wide as long, with one carina, 0.3 times as long as scutellum; scutellum convex, smooth except a few punctures; metanotum with complete median carina but not protruding dorsally; propodeum smooth antero-laterally, reticulate medially and areolate posteriorly, with wide irregular pentagonal areola medially (Fig. 6F); propodeal spiracle round, small and submedially in propodeum.

***Wings*.** Pterostigma elongate subtriangular, its posterior margin straight or slightly convex; vein r issued near middle of pterostigma and oblique; only known species with basal half of 1-R1 distinctly widened; r:3-SR:SR1 = 8:41:34; 1-SR+M narrow and straight; SR1 straight; cu-a medium-sized, postfurcal; 1-CU1:2-CU1 = 1:11; 2-SR:3-SR:r-m = 19:41:14; m-cu postfurcal and slightly curved, subparallel to 1-M; 3-CU1 slightly shorter than CU1b and widened (Fig. 6A). Hind wing: M+CU:1-M (up to m-cu):1r-m = 20:17:10; m-cu distinct, largely unsclerotised and distantly antefurcal.

***Legs*.** Hind coxa smooth and baso-ventrally wide rectangular and not protruding; fore tarsal claws rather robust (other missing); length of femur, tibia, and basitarsus of hind leg 5.5, 12.2, and 10.4 times their width, respectively; hind tibia and basitarsus with rather long setae, hind tibia densely setose, comb at inner apex of tibia absent; fore tarsus 1.5 times as long as fore tibia.

***Metasoma*.** Length of first tergite 2.4 times its apical width, its surface longitudinally costate, its dorsal carinae nearly complete (Fig. 6F); laterope absent; dorsope large and deep, pointed dorsally (Fig. 6F); remainder of metasoma smooth and depressed; hypopygium medium-sized and slightly concave posteriorly; parameres large.

***Colour*.** Blackish chestnut brown; scapus, pedicellus, mandible, legs (but middle and hind coxae, hind tibia, except basally, and hind tarsus dark brown) yellowish brown; palpi (but basally brownish), basal fifth of fore and middle tibiae, and basal 0.4 of hind tibia whitish or pale yellowish; tegulae, remainder of antenna, pterostigma and veins more or less dark brown; wing membrane faintly brownish.

##### Distribution.

Indonesia (Sumatra).

##### Etymology.

Named after the collector of the holotype, the hymenopterist Prof. Dr Jacobus van der Vecht (1906–1992) for his excellent contributions to our knowledge of Hymenoptera (van Achterberg 1992).

#### ﻿ Anamalysia vanhengstumi

Taxon classificationAnimaliaHymenopteraBraconidae

van Achterberg sp. nov.

19E88865-296F-5792-B0D3-B1211383DDB2

https://zoobank.org/74022A0F-DE25-42EB-811D-E60E50365907

Figs 7A, B , 8A–I

##### Type material.

***Holotype***, ♂ (RMNH), “Alysi. 070”, VN [= Vietnam], Ha Giang, Vi Xuyen, Cao Bo Rung TS, 400 m, 10.v.2007, K.D. Long.

##### Description.

***Holotype***, ♂, length of body 3.9 mm, length of fore wing 3.3 mm.

**Figure 7.**
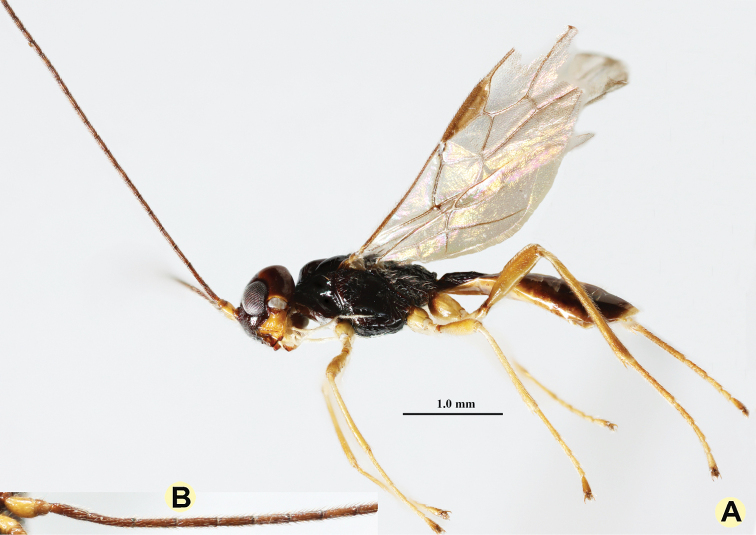
*Anamalysiavanhengstumi* sp. nov., ♂, holotype **A** habitus, lateral aspect **B** detail of basal antennal segments.

**Figure 8.**
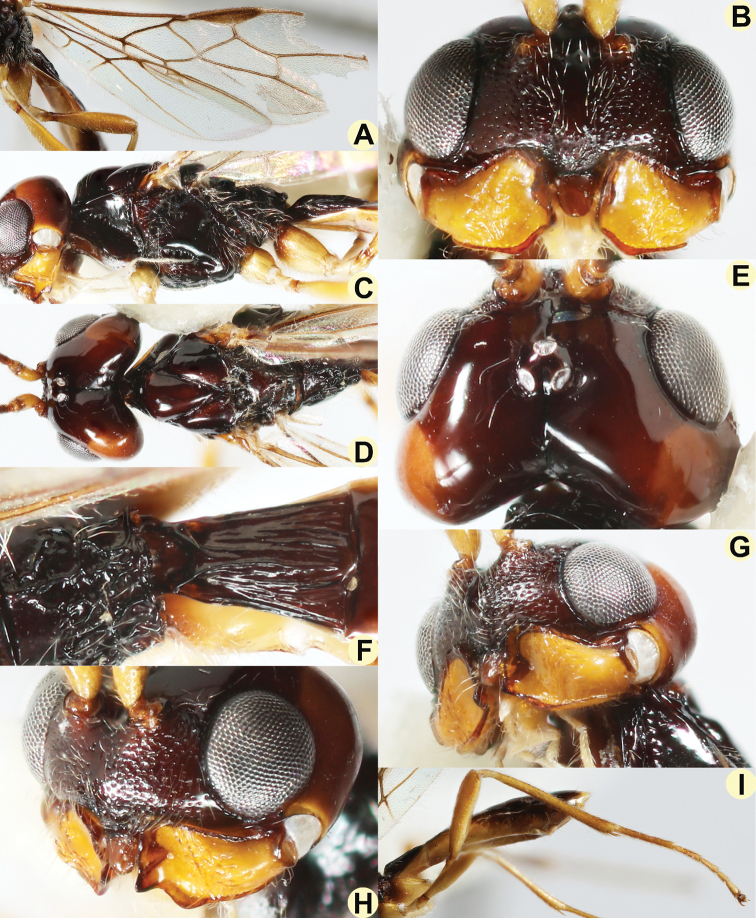
*Anamalysiavanhengstumi* sp. nov., ♂, holotype **A** wings **B** head, anterior aspect **C** mesosoma, lateral aspect **D** head and mesosoma, dorsal aspect **E** head, dorsal aspect **F** propodeum and first metasomal tergite, dorsal aspect **G** mandible, full sight on first tooth **H** mandible, with full sight on third tooth **I** hind leg.

***Head*.** Width of head 2.4 times its median length, deeply depressed medially and largely glabrous dorsally; antenna incomplete, with 29+ segments, setae short and adpressed and seven basal segments strongly shiny, length of third segment 0.9 times as long as fourth segment, length of third and fourth segments 3.9 and 4.6 times their width, respectively (Fig. 7B); length of maxillary palp 1.2 times height of head; eye in dorsal view 1.1 times as long as temple; temple in dorsal view strongly widened behind eyes (Fig. 8E); OOL:diameter of ocellus: POL = 15:4:3; minimum width of face 0.6 times maximum width of head and 1.8 times its height, coarsely punctate, moderately convex, with long setae and medio-ventrally narrowly smooth (Fig. 8B); with oblique groove from antennal socket to eye; clypeus rather robust and largely smooth (Fig. 8B); vertex strongly shiny and weakly convex and strongly depressed behind stemmaticum; anterior tentorial pit covered by mandible; length of malar space 0.1 times basal width of mandible; mandible strongly widened, 1.4 times as long as wide, middle tooth dorsally connected to wide sinuate and up curved lamella from upper corner of mandible, medially coarsely rugose, first tooth part of apical lamella and third tooth medium-sized (Fig. 8G, H).

***Mesosoma*.** Length of mesosoma 1.7 times its height; pronotum with large pronope; antescutal depression absent; side of pronotum largely crenulate medially (except subposteriorly) and posteriorly; epicnemial area with few crenulae; precoxal sulcus absent posteriorly, wide and coarsely crenulate (Fig. 8C); remainder of mesopleuron smooth; episternal scrobe elongate and medium-sized; pleural sulcus finely crenulate dorsally and distinctly crenulate ventrally; mesosternal sulcus wide and coarsely crenulate; metapleuron largely smooth but rugose ventrally; notauli complete, deeply impressed, rather narrow, and smooth; medio-posterior depression absent (Fig. 8D); mesoscutum smooth, glabrous, and its lateral carina complete in front of tegulae; scutellar sulcus deep, about 2.5 times as wide as long, with one carina, 0.3 times as long as scutellum; scutellum slightly convex, smooth except a few punctures; metanotum with complete median carina and rather protruding dorsally; propodeum smooth antero-laterally, reticulate medially and areolate posteriorly, with posteriorly narrowed pentagonal areola medially (Fig. 8F); propodeal spiracle small, round, and submedially in propodeum.

***Wings*.** Pterostigma elongate subtriangular, its posterior margin straight or slightly convex; vein r issued from basal 0.6 of pterostigma and oblique; 1-R1 narrow; r:3-SR:SR1 = 5:22:42; 1- SR+M narrow and nearly straight; SR1 straight; cu-a medium-sized, postfurcal; 1-CU1:2-CU1 = 1:11; 2-SR:3-SR:r-m = 20:22:9; r-m weakly inclivous; m-cu subinterstitial and slightly curved, converging to 1-M; 3-CU1 much longer than CU1b and narrow (Fig. 8A). Hind wing: M+CU:1-M (up to m-cu):1r-m = 20:18:9; m-cu distinct, largely unsclerotised (except basally) and interstitial (Fig. 8A).

***Legs*.** Hind coxa smooth, baso-ventrally rounded, and not protruding; tarsal claws rather robust; length of femur, tibia, and basitarsus of hind leg 4.2, 11.0, and 9.0 times their width, respectively; hind tibia and basitarsus with numerous rather long setae dorsally; hind tibia densely setose and comb at inner apex of tibia absent; fore tarsus 1.4 times as long as fore tibia (Fig. 8I).

***Metasoma*.** Length of first tergite 1.4 times its apical width, its surface longitudinally costate, its dorsal carinae united submedially (Fig. 8F); laterope deep and large; dorsope large, deep, and pointing dorsally; remainder of metasoma smooth and depressed; hypopygium truncate posteriorly (Fig. 7A).

***Colour*.** Black; scapus, pedicellus, mandible, and legs yellow; remainder of antenna dark brown; temple and vertex and metasoma laterally (except first tergite) chestnut brown; palpi ivory; tegulae mainly yellowish brown; pterostigma and most veins brown; wing membrane faintly infuscated.

##### Distribution.

Northern Vietnam.

##### Etymology.

Named after the former director of the National Museum of Natural History (Naturalis) Ronald van Hengstum (1952–2007), who tragically died after a short swim in the North Sea near The Hague. He visited Vietnam during one of the RMNH-IEBR expeditions and was strongly in favour of cooperation with our Vietnamese counterparts.

## Supplementary Material

XML Treatment for Anamalysia

XML Treatment for Anamalysia idiastimorpha

XML Treatment for Anamalysia knekosoma

XML Treatment for Anamalysia mellipes

XML Treatment for Anamalysia transversator

XML Treatment for Anamalysia triangulum

XML Treatment for Anamalysia urbana

XML Treatment for Anamalysia vandervechti

XML Treatment for Anamalysia vanhengstumi
